# A Novel Variable Volume Capillary Microgripper for Micromanipulation in Aqueous Media

**DOI:** 10.3390/mi16060633

**Published:** 2025-05-27

**Authors:** Enrique Mancha-Sánchez, Andrés J. Serrano-Balbontín, Inés Tejado, Blas M. Vinagre

**Affiliations:** Escuela de Ingenierías Industriales, Universidad de Extremadura, 06006 Badajoz, Spain; ajserranob@unex.es (A.J.S.-B.); bvinagre@unex.es (B.M.V.)

**Keywords:** capillary microgripper, micromanipulation, aqueous environment, variable volume, silicone oil, image-based control, two-photon polymerization, surface tension

## Abstract

This study presents a novel capillary microgripper for manipulating micrometer-sized objects directly within aqueous environments. The system features dynamic, vision-based feedback control of a non-volatile silicone oil droplet volume, enabling precise adjustment of the capillary bridge force for the adaptable capture of varying object sizes. This approach ensures extended working time and stable operation in water, mitigating the issues associated with evaporation common in other systems. COMSOL Multiphysics simulations analyzed capillary bridge formation. Experimental validation demonstrated successful different object shapes and sizes capture in an aqueous environment and further explored active release strategies necessary due to the non-volatile fluid, confirming the system potential for robust underwater micromanipulation.

## 1. Introduction

Manipulating objects at micro and nano scales is crucial for applications ranging from semiconductor manufacturing and microelectromechanical system (MEMS) assembly to medical device fabrication and biological structure handling. However, working in these dimensions requires overcoming the physical and technical barriers inherent to extreme miniaturization. Manipulating structures at the submillimeter scale poses critical challenges, such as the predominance of surface adhesion forces over gravitational forces, the mechanical fragility of components and the need for nanometer precision in their positioning. These challenges limit the effectiveness of conventional techniques, such as mechanical grippers which risk damaging fragile components, or vacuum systems that struggle with porous objects [1,2], especially in the wet or biological environments critical for many miniaturization tasks. Devices utilizing the surface tension forces generated by the presence of a capillary bridge offer several advantages over other methods, including enhanced precision in manipulation and reduced damage to the manipulated components.

Capillary forces arise from the surface tension of liquid bridges between microscale objects. They have become a key to non-destructive micromanipulation in fields such as microassembly, biomedical engineering, and microelectronics [3]. Early theoretical models, such as those derived from the Young–Laplace equation, established the relationship between liquid bridge geometry, surface tension, and the resultant adhesive forces [4]. Translating these principles into functional microgrippers needed advancements in microfabrication and dynamic control. For instance, it has been demonstrated that setting parameters such as liquid volume [5,6], contact angle hysteresis [3,7], and substrate hydrophobicity [8] could modulate gripping forces, enabling adaptive pick-and-place operations. One of the most compelling advantages of capillary microgripping is its passive nature. Unlike traditional grippers that rely on complex mechanical or electrical systems, capillary microgrippers require no external power to generate adhesive forces [9]. In addition to non-destructive manipulation, capillary forces offer an intrinsic advantage of self-alignment. The surface tension of the liquid bridge tends to center the object relative to the microgripper tip, significantly simplifying the precise positioning requirements during capture [10,11,12]. This makes them particularly well suited for applications in delicate environments, such as handling living cells or assembling MEMS. In this sense, challenges remain, for example, the evaporation of the liquid bridge can limit the operational time [13], while the unintended adhesion of particles or contaminants can compromise performance [14].

Different mechanisms for capillary microgrippers can be found in the literature. In [6], the microgripper uses a piston slider to form a water droplet at the tip of a nozzle, by an on/off control mechanism. A double-nozzle capillary force gripper is presented in [7], which uses two nozzles to form droplets through a combination of capillary action and on/off control of a diaphragm pump for droplet formation. Other studies have employed numerical simulations based on the Young–Laplace equation to analyze the equilibrium shapes of liquid bridges and their dependence on parameters such as surface tension, contact angle, and volume of the liquid [8,15]. Efforts have also been made to overcome fabrication complexities in capillary grippers through hybrid multiscale manufacturing methods [16]. Additionally, computational fluid dynamics (CFD) and finite element analysis (FEA) have been utilized to optimize the geometry of microgrippers and to study the dynamics of liquid bridge formation and rupture [2,5]. Theoretical frameworks have provided critical insights into the scaling laws governing capillary forces at the microscale [17,18].

Micromanipulation into aqueous environment can be crucial for applications like in vitro fertilization [19] and biomedical microrobotics, capable of performing minimally invasive interventions within the human body [20]. A particularly challenging yet promising application in this domain involves the precise delivery and release of microscale therapeutic payloads, diagnostic agents, or microrobots from the tip of a catheter directly within dynamic physiological environments, such as the bloodstream. This specific scenario motivates the development of robust grippers capable of secure object retention during transit and controlled release at a target site, while operating effectively within an aqueous medium. For conventional capillary methods, this is a fundamental challenge. Using water as the bridging fluid is ineffective within an aqueous medium due to the lack of interfacial tension. Effective capillary gripping requires significant interfacial tension between the bridging fluid and the surrounding medium. To address this, we employ silicone oil as the bridging fluid. Its interfacial tension with water (approximately 36 mN/m at room temperature [21]) is sufficient, when combined with object buoyancy, to generate the necessary capillary forces for stable object retention, despite being lower than the air–water surface tension (approximately 72 mN/m). This use of a biocompatible [22], non-volatile oil enables capillary gripping principles to be effectively applied within aqueous environments.

The objective of this work is to design, fabricate, and validate a microgripper that uses capillary forces to grab and release objects in an aqueous environment by combining three key features: (1) droplet volume control, enabling adaptation to a wider range of object sizes; (2) operation within water, a crucial medium for some applications; and (3) the use of a non-volatile fluid (silicone oil) for extended working time and stable operation in an aqueous environment. While these individual elements exist in various contexts, their synergistic integration into a single microgripper specifically designed and validated for adaptable micromanipulation within aqueous environments represents the core novelty of this work.

The article is structured as follows. Section 2 describes the mathematical model and simulations performed to analyze the capillary bridge formation, and details the design, fabrication, and experimental setup of the microgripper, including the fluid delivery and image processing components. Section 3 presents and discusses the experimental results obtained for droplet control and microrobot capture, focusing on the challenges of object release. Finally, Section 4 concludes the article by summarizing the key findings and outlining directions for future research.

## 2. Materials and Methods

This section contains the materials and methods involved in the design and fabrication of the microgripper.

### 2.1. Theoretical Background of Capillary Bridging

**Figure 1:**
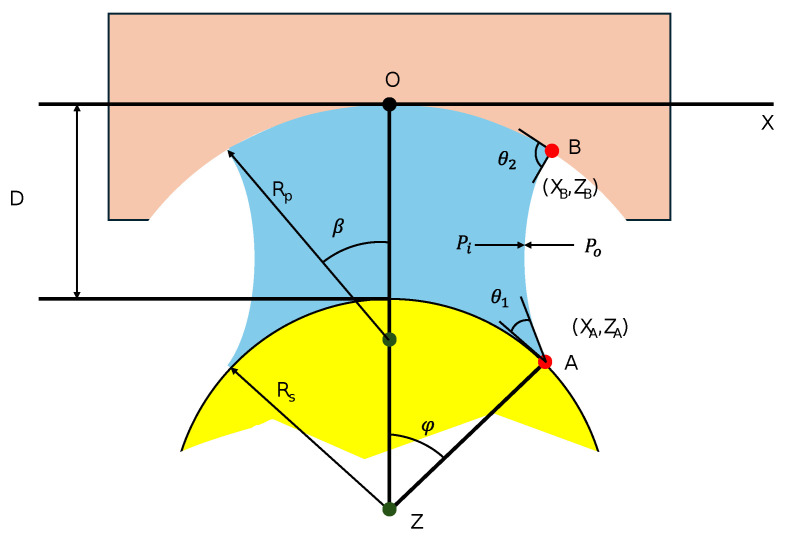
Geometry of the axisymmetric capillary bridge formed between a concave gripper (radius $R_{p}$) and a spherical object (radius $R_{s}$). Key parameters shown include the separation distance (*D*), internal ($P_{i}$) and external ($P_{o}$) pressures, contact angles ($\theta_{1}$, $\theta_{2}$), half-filling angles (ϕ, β), and contact point coordinates (*A*, *B*).

The gripping mechanism relies on the capillary force generated by a liquid bridge formed between the microgripper tip and the target object (Figure 1). In an axisymmetric configuration, the shape of the liquid meniscus, X(Z), is governed by the pressure difference ($\Delta P=P_{o}-P_{i}$) between the surrounding fluid (exterior, $P_{o}$) and the bridging fluid (interior, $P_{i}$). This relationship between the interfacial curvature and pressure difference is fundamentally described by the Young–Laplace equation [4].

The resulting capillary force ($F_{cp}$) acting on the object generally comprises two components: a force due to the pressure difference acting over the contact area ($F_{p}\propto \Delta PX_{A}^{2}$) and a force due to the interfacial tension (γ) acting along the three-phase contact line ($F_{s}\propto \gamma X_{A}\sin\left(\varphi +\theta_{1}\right)$) [8,18].

The geometry and stability of the bridge, and consequently the capillary force, depend critically on factors including the radii of the interacting surfaces ($R_{s},R_{p}$), the separation distance (*D*), the volume of the bridging liquid (*V*), the interfacial tension (γ) between the two fluids, and the contact angles ($\theta_{1},\theta_{2}$) at the liquid–solid interfaces [17,18].

Calculating the exact bridge profile and forces typically involves solving the non-linear Young–Laplace differential equation with appropriate boundary conditions derived from the system geometry and contact angles. While this analytical framework provides the theoretical foundation for capillary gripping, the specific numerical simulations performed in this work utilized the phase field method within COMSOL Multiphysics 6.2 (detailed in Section 2.2) to model the two-phase fluid dynamics and interface formation.

### 2.2. Simulations

To investigate the capillary bridge dynamics in the oil–water system, the governing equations for two-phase immiscible flow were solved numerically using the finite element method (FEM). All simulations were performed using a 2D axisymmetric model, representing a 2D cross-section revolved around the central axis to define the 3D geometry. This approach assumes axial symmetry in the gripper tip, the object, and the resulting capillary bridge, allowing for computationally efficient yet accurate modeling of the primary axial forces and interface dynamics. The model developed represents the microgripper, the captured object, an ellipsoid with semi-axes a=0.8 mm, b=1 mm, the surrounding aqueous environment, water, and the initial volume of bridging fluid, silicone oil. The microgripper geometry was modeled based on its design dimensions (Section 2.3), featuring a concave tip profile. The computational domain included a region representing the surrounding water environment.

#### 2.2.1. Governing Equations

The simulation employed the phase field method to track the diffuse interface between the water (Fluid 1) and silicone oil (Fluid 2). The evolution of the phase field variable (ϕ, ranging from −1 in water to +1 in oil) is governed by the Cahn–Hilliard equation:

(1) $$\frac{\partial \phi }{\partial t}+u\cdot \nabla \phi =\nabla \cdot \left(M\nabla \psi \right)$$

(2) $$\psi =-\nabla \cdot \left(\epsilon^{2}\nabla \phi \right)+\left(\phi^{2}-1\right)\phi$$

where u is the fluid velocity, ψ is the phase field help variable, $M=\gamma \lambda /\epsilon^{2}$ is the mobility (related to mobility parameter γ), λ is the mixing energy density, and ϵ is the interface thickness parameter. The interfacial tension (σ) is related to these parameters by $\sigma =\left(2\sqrt{2}/3\right)\left(\lambda /\epsilon \right)$. The volume fractions ($V_{f1},V_{f2}$), density (ρ), and dynamic viscosity (μ) throughout the domain are functions of ϕ:

(3) $$\begin{array}{c} V_{f1}=\left(1-\phi \right)/2,\;V_{f2}=\left(1+\phi \right)/2 \end{array}$$

(4) $$\begin{array}{c} \rho =\rho_{f1}V_{f1}+\rho_{f2}V_{f2} \end{array}$$

(5) $$\begin{array}{c} \mu =\mu_{f1}V_{f1}+\mu_{f2}V_{f2} \end{array}$$

The specific fluid properties used are $\rho_{f1}=1000$ kg/m^3^, $\mu_{f1}=0.001$ Pa·s (water); $\rho_{f2}=850$ kg/m^3^, $\mu_{f2}=0.017$ Pa·s (silicone oil); and σ=36 mN/m.

The fluid motion is described by the Navier–Stokes equations for incompressible flow, including the surface tension force ($F_{st}$) derived from the phase field chemical potential (*G*):

(6) $$\rho \left(\frac{\partial u}{\partial t}+\left(u\cdot \nabla \right)u\right)=\nabla \cdot \left[-pI+\mu \left(\nabla u+{\left(\nabla u\right)}^{T}\right)\right]+F_{st}+\rho g$$

(7) ∇·u=0

where *p* is the pressure and g is the gravitational acceleration. The surface tension force is calculated as $F_{st}=G\nabla \phi$, where $G=\lambda \left[-\nabla^{2}\phi +\phi \left(\phi^{2}-1\right)/\epsilon^{2}\right]$.

To couple the phase field physics with the fluid dynamics, the surface tension force is included in the Navier–Stokes equations as a force that depends on the chemical potential of the system and the gradient of the phase field variable:

(8) $$\rho \frac{\partial u}{\partial t}+\rho \left(u\cdot \nabla \right)u=\nabla \cdot [-pI+\mu (\nabla u+{\left(\nabla u\right)}^{T})]+F_{st}+\rho g$$

(9) ∇·u=0

where ρ is the density, μ is the dynamic viscosity, u is the velocity, *p* is the pressure, and g is the gravity vector. $F_{st}$ is the surface tension force that acts on the fluid interface, also known as the interfacial tension. At the phase field interface, the diffuse interface representation allows the surface tension to be calculated by

(10) $$F_{st}=G\nabla \phi$$

where ϕ is the previously annotated phase field parameter, and *G* is the chemical potential:

(11) $$G=\lambda \left[-\nabla^{2}\phi +\frac{\phi (\phi^{2}-1)}{\epsilon^{2}}\right]=\frac{\lambda }{\epsilon^{2}}\psi$$

This way, the phase field surface tension is calculated as a force distributed over the interface using only ψ and the gradient of the phase field variable. In this way, the phase field interface can be included in the fluid dynamics, allowing to couple the interface between two immiscible fluids and see its behavior.

#### 2.2.2. Initial and Boundary Conditions

The initial conditions established correspond to the initial volume of silicone oil. This volume will allow the desired capillary bridge to be generated between the captured object and the microgripper. The boundary conditions imposed on the model are the silicone oil placed between the two concave surfaces, the open boundary, and the so-called “wetted wall” condition. The “open boundary” condition is applied to the outer region of the geometry where water is allowed to freely enter and exit the domain. The “wetted wall” feature is used for solid walls in contact with a fluid interface. In this case, it was set on the walls of the microgripper and the captured object. It sets the velocity component normal to the wall to zero, i.e., $u\cdot n_{wall}=0$, and adds a friction force to the contour $F_{fr}=-\left(\mu /\beta \right)u$. Here, β is the slip length. In our case, the boundary condition of the θ contact angle, i.e., the angle between the walls and the fluid interface, was specified. The contact angle was set to $45^{\circ }$, and the slip length was equal to the mesh element size *h*.

#### 2.2.3. Mesh and Simulation

A 2D axisymmetric finite element mesh consisting of approximately 12,900 triangular elements was generated, with refinement near the expected fluid interface. Two primary simulations were performed. First, a simulation was run to observe droplet formation dynamics. This was initialized defining a inlet volume of silicone oil (Fluid 2). The simulation was run over time to visually confirm that the initial oil volume form into a stable drop. Second, a simulation was conducted specifically to determine the capillary force exerted on the object. This simulation formed a droplet over the object to form a stable capillary bridge. The net capillary force exerted vertically on the object was then calculated within COMSOL by integrating the pressure and viscous stress tensor components over the object wetted surface boundary.

### 2.3. Microgripper Fabrication

**Figure 2:**
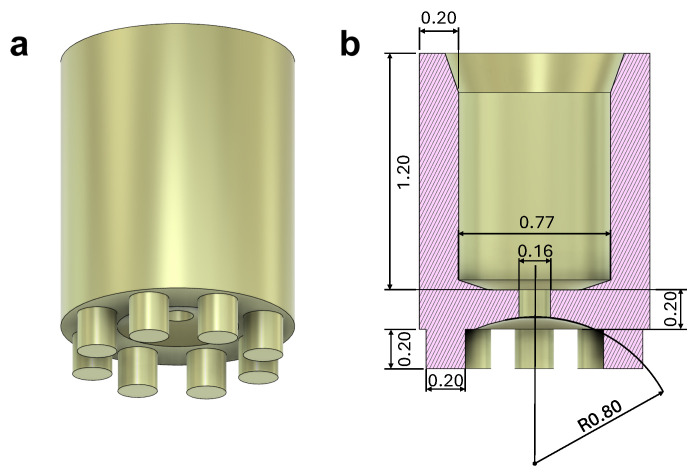
Microgripper CAD design, (**a**) overall view, (**b**) cross-sectional view (units in mm).

The global design and the working principle of the microgripper is presented here. The microgripper design is determined by two fundamental aspects: its size and the fluid delivery system. The size of the microgripper is dictated by the dimensions of the body to be captured and released. Given that the object range to be captured is between 0.1 and 2 mm, the gripper dimensions must be adjusted accordingly to enable the capture and release of this size of objects. On the other hand, the fluid delivery system determines the type of connection required between the tube/syringe and the microgripper. In Figure 2, the design of the microgripper can be seen. It has a cylindrical shape with a 1.17 mm outside diameter, 770 μm inner diameter, and 1.6 mm height. The bottom hole where fluid is released is 160 μm in diameter. As can be seen in Figure 2, there are attached columns in the perimeter of the bottom plane of the microgripper. These structures prevent the outlet hole of the microgripper from being obstructed by the object to be captured. Similarly, during the release process, the object must allow the complete removal of the fluid without causing blockage in the microgripper.

**Figure 3:**
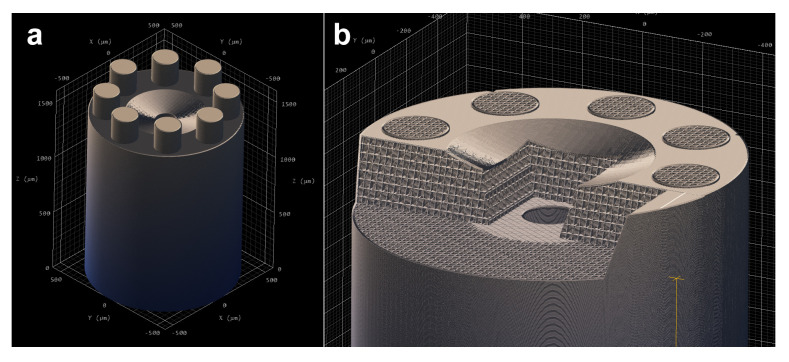
(**a**) Nanoscribe slicing process; (**b**) detailed section of the sliced internal scaffold.

Conventional additive manufacturing processes are incapable of producing the submillimeter features of this microgripper. Two-photon polymerization printing provides the resolution required to fabricate these intricate structures. Microgripper was printed using Nanoscribe Photonics Professional GT2 with the dip-in laser lithography (DiLL) configuration. IP-S Resin was used with a 25× objective in an indium tin oxide (ITO) coated glass substrate. The shell and scaffold writing strategy with tetrahedral scaffold type was used. Special layer overlap configuration of 2 μm was used to enhance adhesion between the microgripper layers, preventing potential fluid leakage. Figure 3 shows its slicing view. The printing time was near 2 h. The microgripper was submerged in propylene glycol monomethyl ether acetate (PG-MEA) for 24 h and then cleaned in isopropyl alcohol for 2 h.

### 2.4. Experimental Setup

**Figure 4:**
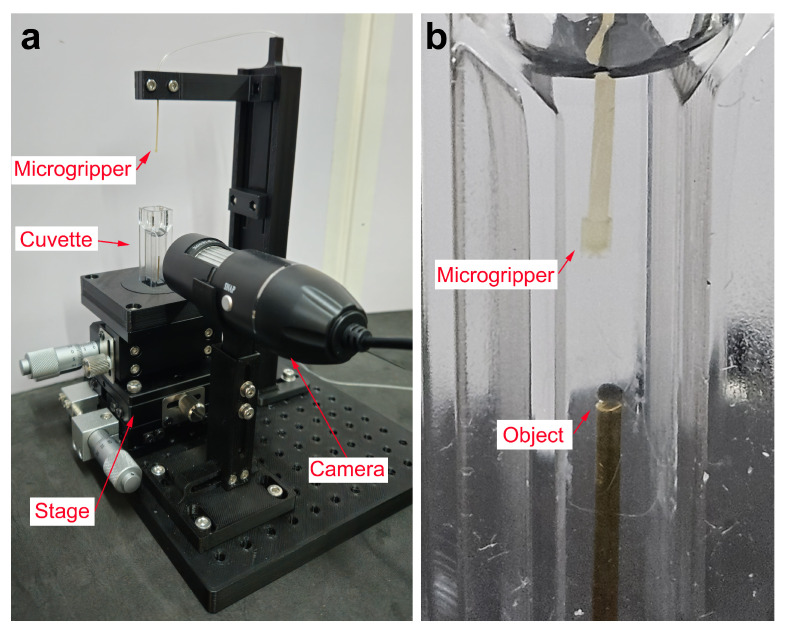
Experimental setup with the microgripper. (**a**) Overall view and (**b**) detailed view of the microgripper within the water-filled cuvette.

The microgripper is placed as shown in Figure 4a. The microgripper is retained by a two-component system designed to provide sufficient grip for stability while minimizing fluid flow restriction. The system translates freely along its supporting column. The microgripper is submerged in a water-filled spectrophotometer cuvette to maximize optical clarity. In this cuvette is placed the object to be captured. The cuvette is mounted on a 3-axis stage, facilitating the precise micropositioning of the submerged target object. For visualization of the process, a camera focused on the cuvette is employed. This camera is attached to a four-degree-of-freedom system, allowing for precise positioning and maintaining focus on object. All these components are placed in a 15×15 cm^2^ breadboard. In Figure 4b, it can be seen a detailed view of the setup with the microgripper immersed in the cuvette with water and one of the object to be captured.

**Figure 5:**
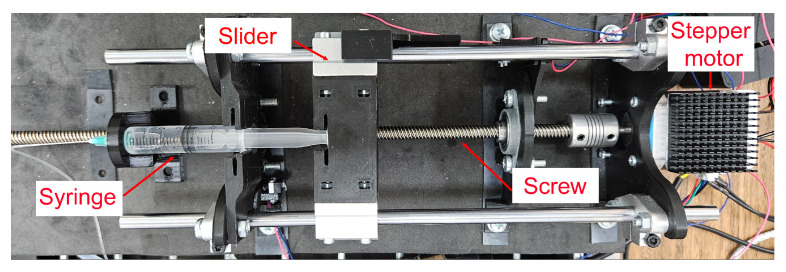
Picture of the syringe pump.

For the oil injection system through the microgripper, a syringe pump was designed, driven by a stepper motor and controlled via an Arduino (Figure 5). This pump actuates the plunger of a syringe, which, connected to the microgripper via a tube, enables fluid delivery. The system comprises three main components: the actuation mechanism, the motor, and the control system.

**Figure 6:**
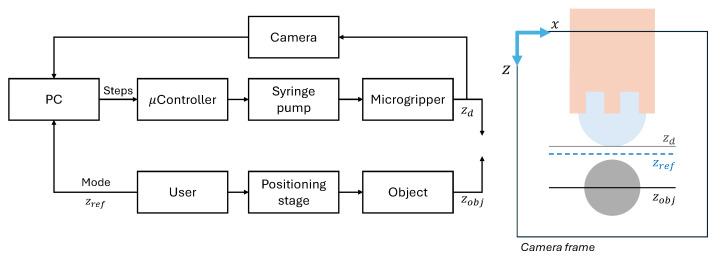
Schematic of the experimental setup and control system. (**Left**) Block diagram illustrating the vision-based feedback loop. The PC processes camera input to determine the droplet edge position ($z_{d}$) compares it to the reference ($z_{ref}$) and sends step commands to the microcontroller driving the syringe pump. User inputs and positioning stage control are also depicted. (**Right**) Definition of key vertical positions within the camera coordinate frame: measured droplet edge ($z_{d}$), target reference ($z_{ref}$), and object position ($z_{obj}$).

The overall system architecture and vision-based control loop are illustrated in Figure 6. The system comprises several key components:

- Actuation mechanism:A syringe pump injects oil into the microgripper via a connecting tube. It utilizes a threaded rod to drive the syringe plunger, allowing for controlled fluid injection or retraction based on the rod rotational direction. The syringe barrel is fixed, and the plunger is attached to a sliding carriage moving along support rods. Limit switches define the travel range.
- Motor and feedback: A stepper motor drives the threaded rod. An AS5600 (ams OSRAM AG, Austria) magnetic encoder provides rotational feedback for precise control.
- Control unit: An Arduino DUE serves as the main controller, processing inputs from the limit switches, magnetic encoder, and managing the DRV8825 (Texas Instrument, Dallas, TX, USA) motor driver (mounted on a custom PCB). An Arduino UNO is used solely to power the motor encoder.
- Fluidic components: A 5 mL syringe with a Luer Lock connection is employed. A 21G needle connects the syringe to a flexible tube with inner diameter (ID) of 0.5 mm, outer diameter (OD) of 1 mm, and a length of approximately 1.5 m. This specific choice of long, thin, and flexible tubing is dictated by the requirements of the primary envisioned application: enabling the microgripper to be guided through the lumen of a long medical catheter to reach internal target sites within the body. A rigid tube (ID: 0.38 mm, OD: 0.77 mm) is press-fit into the microgripper inlet and connected to the flexible tube.
- Control algorithm:
  - The Arduino DUE program controls the stepper motor steps based on feedback from the magnetic encoder (via I2C protocol) and incorporates limit switch sensor logic. It receives high-level commands (steps, direction, and speed) via serial communication.
  - A Python 3.10 program running on a PC manages the overall process, including visualization, real-time vision system analysis (using an attached camera), and sending commands to the Arduino for motor control. Its primary objective in the control loop is to adjust the syringe piston movement precisely to maintain the droplet lower apex aligned with a defined reference line in the captured image.

### 2.5. Image Processing

**Figure 7:**
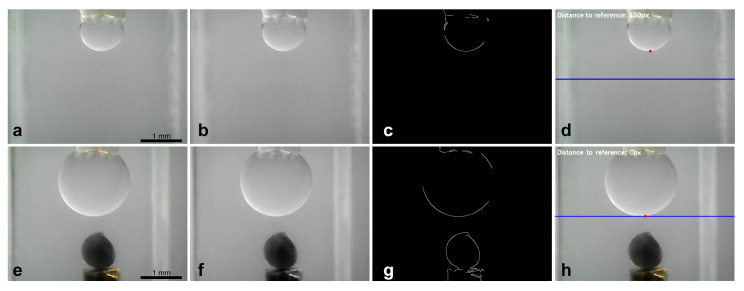
Sequence of image segmentation, (**a**) from original image take by the camera (**b**) to applying grayscale and blur and (**c**) to Canny edge detector, and (**d**) final with red dot as the lower droplet position and blue line as the reference. (**e**–**h**) Sequence of image segmentation for target object identification.

Vision-based feedback control of the oil droplet volume was managed using a custom Python program utilizing the OpenCV library. This program aimed to establish and maintain a desired vertical position of the droplet lower apex relative to a defined reference point in the camera field of view. The process commenced with image acquisition from the designated camera (Figure 7a). Each captured frame underwent pre-processing, starting with conversion to grayscale to reduce computational complexity. A Gaussian blur (using a 5×5 kernel) was applied to mitigate image noise, which could otherwise interfere with edge detection (Figure 7b). Subsequently, the Canny edge detection algorithm was employed to identify sharp intensity gradients corresponding to object boundaries, including the droplet interface (Figure 7c). To specifically locate the droplet lower apex, a Region of Interest (ROI) was defined vertically below the known position of the microgripper tip in the image. Within this ROI, contours corresponding to detected edges were extracted. The contour representing the droplet interface was identified based on proximity to the expected location, and its lowest vertical point (minimum y-coordinate in image coordinates) was determined. This point represented the measured droplet edge position $z_{d}$ as shown in Figure 7d. The control error *e* was calculated as the difference between the desired reference position (setpoint, $z_{ref}$) and the measured position ($z_{d}$). Based on this error, the Python program commanded the Arduino DUE controlling the stepper motor via serial communication. If the measured edge was above the reference, the motor was instructed to advance the syringe plunger (injecting oil); if below, the motor retracted the plunger. This closed-loop process adjusted the droplet volume to maintain the target edge position. A similar image processing sequence (grayscale conversion, Gaussian blur, and Canny detection) was used to initiate the search for the target object within the cuvette once the droplet volume was stabilized. Upon object identification, the micropositioning stage was manually raised to bring the object into contact with the stabilized droplet. Object release involved commanding the syringe pump to retract a significant volume of fluid, aiming to break the capillary bridge.

To relate the pixel measurements to physical dimensions, the system was calibrated by imaging a known scale placed at the focal plane. The camera used provided a resolution of 640×480 pixels. Calibration determined the image scale to be approximately 6.9 μm per pixel in the object plane. This spatial resolution dictates the theoretical limit of precision for detecting changes in the droplet edge position using this imaging setup.

## 3. Results and Discussion

This section details the results obtained for simulations and the automated control of droplet volume for object capture.

### 3.1. Simulation

**Figure 8:**
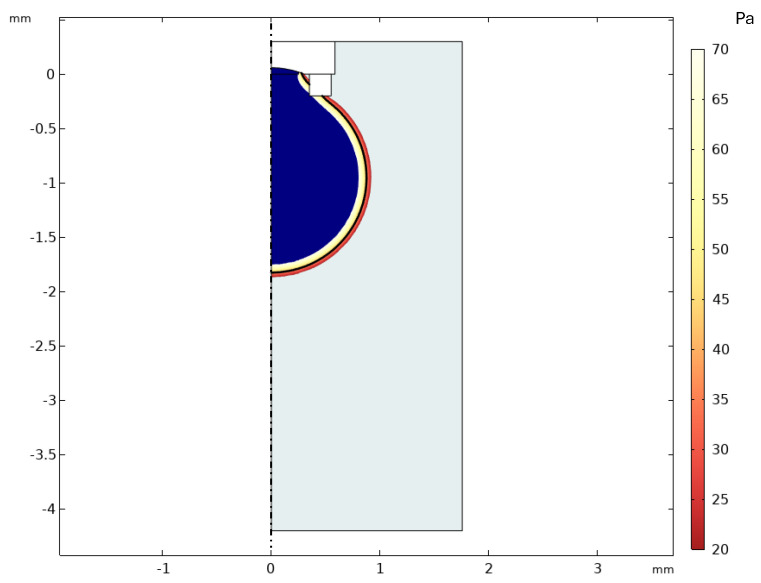
COMSOL 2D-axisymmetric simulation result showing stable silicone oil droplet formation from the microgripper tip in water, visualized by the phase field contours. The color scale indicates the pressure field.

A simulation run initialized with a predefined volume of silicone oil confirmed the formation of a stable pendant droplet from the microgripper tip. Figure 8 illustrates the droplet shape after the simulation reached a quasi-steady state following the initialization of the oil volume. The simulation domain represents a cross-section of the cuvette, with the microgripper geometry depicted at the top. The resulting silicone oil droplet (dark blue region) is shown extending downwards into the surrounding water (light grey). The color contours overlaid on the droplet interface (transitioning from yellow to red) represent the magnitude of the pressure jump ($\Delta P=P_{o}-P_{i}$) across the oil–water interface as calculated by the phase field model.

**Figure 9:**
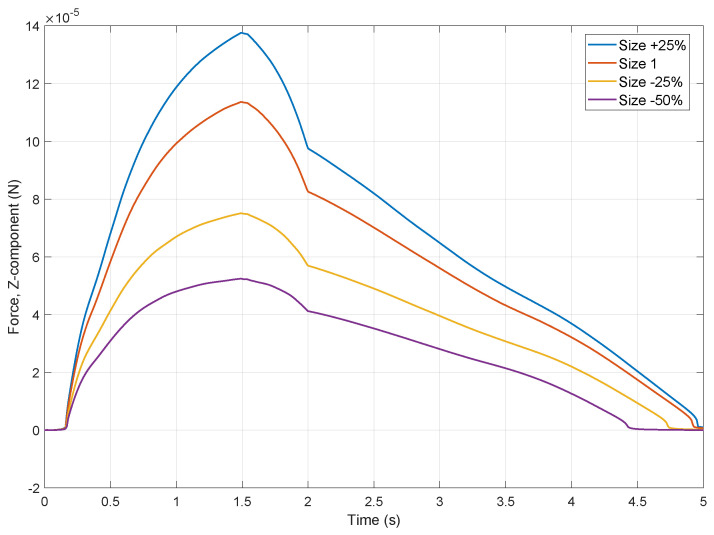
Effect of object size on the simulated vertical capillary force (Z-component) over time. Curves represent object sizes of +25%, baseline (“Size 1”), −25%, and −50% relative to the baseline size, simulated with identical initial droplet volumes. Retraction phase initiated around t=1.5 s, and detachment occurs around t=4.5 s.

To further investigate the system dynamics, simulations were performed varying the size of the object while keeping the initial droplet volume constant. Figure 9 plots the calculated vertical capillary force (Z-component) exerted on the object over time for four different object sizes, relative to a baseline (“Size 1”). All simulations exhibit a similar temporal profile: the force rapidly increases during bridge formation, reaches a peak value around *t* = 1.5–1.8 s, shows a distinct change in slope around t=2 s, and subsequently decreases until the bridge detaches, when force approaches zero, around *t* = 4.5–5 s. The force magnitudes are in the microNewton range, consistent with the expected capillary interactions. Crucially, the results demonstrate a clear correlation between object size and the maximum achievable capillary force. The peak attractive force significantly increases with larger object size, ranging from approximately 50 μN for the smallest object (−50% size) to about 140 μN for the largest object (+25% size). This finding aligns with the theoretical predictions, as a larger object surface generally allows for a greater contact area and contact line length for a given fluid volume, strengthening the capillary bridge through both pressure difference and surface tension contributions. While this 2D axisymmetric approach provides fundamental insights, it is important to note that it does not capture potential non-axisymmetric effects that might arise with highly irregular object geometries or asymmetrical interactions. While a full 3D model could address these specific cases, it would also involve a substantially greater computational effort.

### 3.2. Droplet Control

To automate, repeat, and predict the object capture process, precise control over the generated droplet volume is essential. As previously described, the droplet position, and consequently the volume, is determined through image segmentation. Controlling the droplet volume ensures sufficient adhesion force for reliable object capture without causing damage.

**Figure 10:**
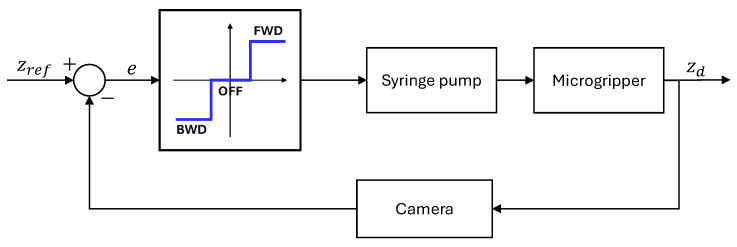
Control loop schematic for droplet edge position regulation. FWD: advance, BWD: retract, OFF: no action, error (*e*), reference setpoint ($z_{ref}$), and measured position ($z_{d}$).

**Figure 11:**
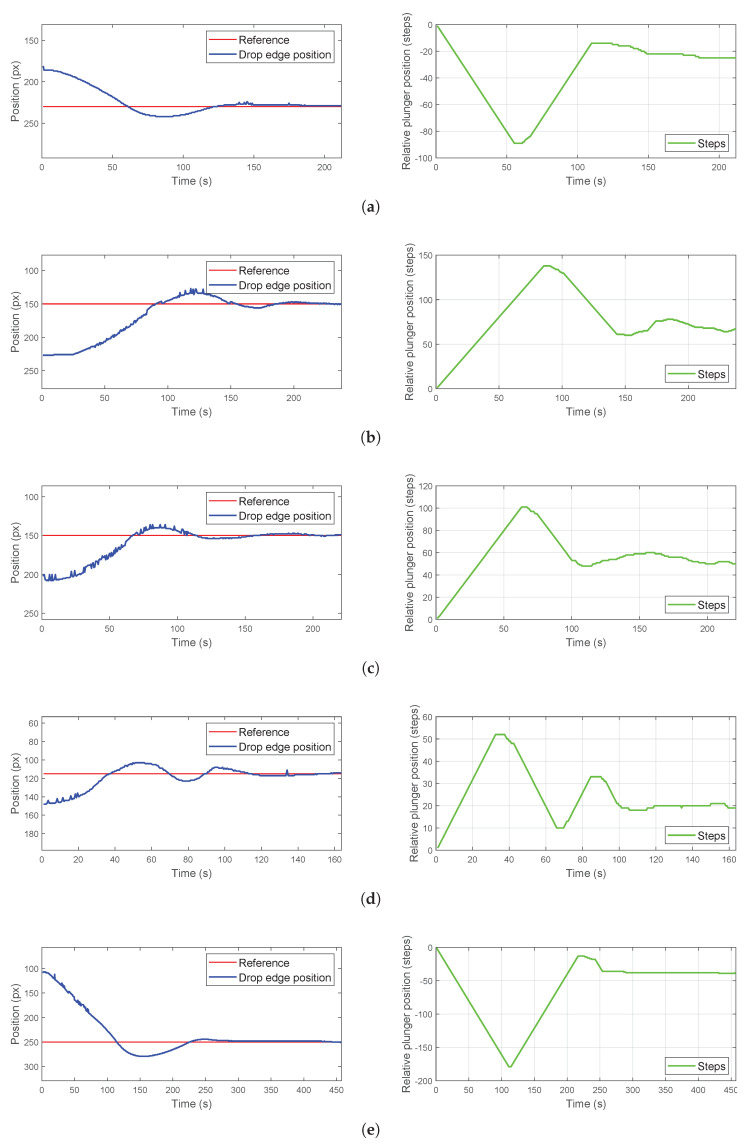
Experimental results of the drop edge position control system. Vertical position of the drop edge (blue line), reference position (red line) and control action, represented as the relative plunger position (green line). For each experiment different references and initial positions were tested: (**a**) 230–181 px, (**b**) 150–227 px, (**c**) 150–202 px, (**d**) 115–148 px, (**e**) 250–108 px.

A closed-loop on/off control strategy with a deadband was employed to regulate droplet position. The controller determines the syringe pump action (advance, retract, and no action) based on the error (*e*) between the reference setpoint ($z_{ref}$) and the measured position ($z_{d}$) obtained from the camera feedback (Figure 10). The droplet edge position was acquired every 500 ms via image segmentation as described previously, and the resulting data were used to drive an Arduino-controlled motor. If the droplet edge was located above the reference position, a command signal was sent to the Arduino to advance the syringe pump plunger with one step. Conversely, if the edge was below the reference, the motor was driven in the opposite direction to retract the plunger. A deadband of 5 px was implemented to account for slow droplet formation and ensure stability; when the droplet edge position fell within the defined deadband range, motor actuation was halted. Different experiments were performed to demonstrate the effectiveness of this control. These experiments explored varying initial droplet positions and target reference values. For each experiment, the corresponding figures illustrate the target reference, the measured droplet edge position with the tolerance range, and the relative plunger displacement. The experimental results are presented in Figure 11. The observed settling time of approximately 200 s, depending on the experiment, is primarily attributable to the hardware configuration imposed by the envisioned catheter-based application. The connecting tube, approximately 1.5 m long with a 0.5 mm internal diameter, is designed for flexibility and to navigate lumens. Such dimensions inherently introduce significant fluidic resistance and compliance, slowing pressure propagation and volumetric response at the droplet as confirmed by the much faster response times observed experimentally with shorter, stiffer tubing.

The performance evaluation of the control system reveals a complex interplay between the reference values and control efficacy (Table 1). As anticipated with on/off control, oscillations are inherent [23], and their characteristics, quantified by oscillation amplitude and period, alongside the steady-state error ($e_{ss}$) and reference normalized integral square error (ISE), serve as primary indicators of system behavior. Across varying reference positions, significant performance disparities were observed. Experiment targeting a reference of 115 px demonstrated a comparatively balanced performance, exhibiting moderate oscillation amplitude and a reasonable steady-state error, while experiments at higher references, particularly 250 and 150 px, showed increased oscillation amplitudes and elevated error integral values.

A key distinction of the present microgripper from previously reported capillary microgrippers lies in its variable droplet volume. While existing designs often rely on fixed droplet volumes dictated by device capabilities (e.g., nozzle size or fluid dispensing mechanism) [24], our system allows for dynamic adjustment of the dispensed volume, enabling adaptation to a wider range of target object dimensions. This variable volume capability is crucial for achieving consistent and successful capture across a variety of object sizes and shapes, as modulation of the droplet volume directly influences the capillary bridge force [25]. Furthermore, the use of a non-volatile fluid allows for an extended working time, facilitating precise droplet volume adjustment. In contrast to our approach, other studies have explored the use of volatile fluids. Evaporation in these systems is a double-edged sword. While it can lead to droplet loss and instability during operations, as reported in [6], it has also been leveraged as a means of object release. The controlled evaporation of the droplet can alter the contact conformity between the droplet and the object, leading to a reduction in capillary forces and facilitating detachment [5]. However, in our system, the non-volatility, while advantageous for precise volume control, presents a challenge, as the residual oil film can cause object adhesion to the microgripper, complicating release as will be seen in the next section.

### 3.3. Experiments for Capturing and Releasing

**Figure 12:**
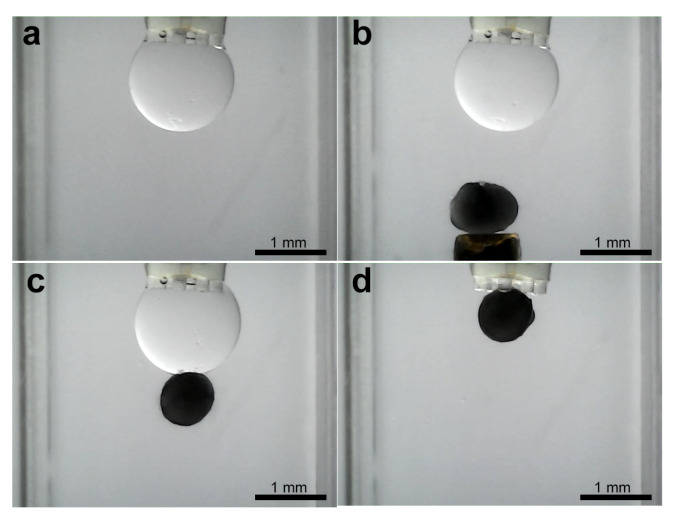
Sequence of capturing of ellipsoidal object. (**a**) Droplet stabilization, (**b**) object reaching, (**c**) object capturing, and (**d**) liberation try.

**Figure 13:**
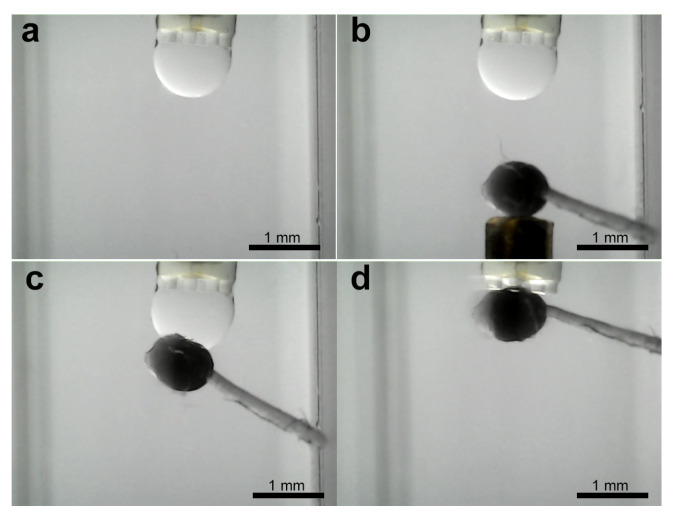
Sequence of capturing of ellipsoidal object with a tail structure. (**a**) Droplet stabilization, (**b**) object reaching, (**c**) object capturing, and (**d**) liberation try.

**Figure 14:**
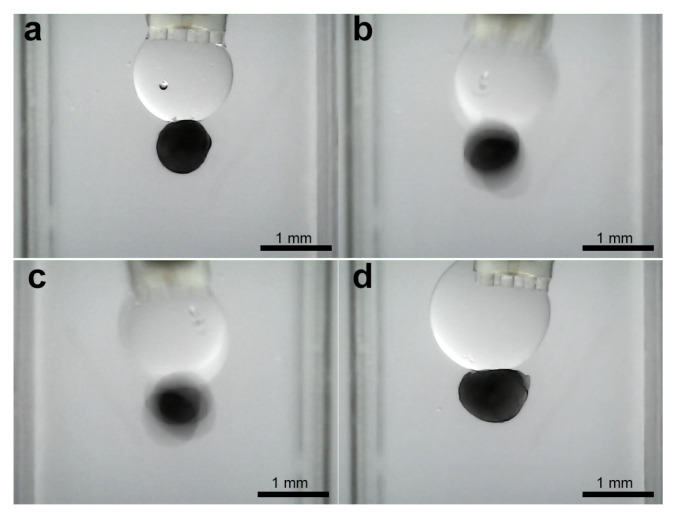
Sequence of attempts to release the object from the droplet formed. (**a**) Object successfully captured, (**b**) first try to release the object, (**c**) next try to release the object, and (**d**) object still captured.

Subsequent to the stabilization of the droplet volume, the object capture process is initiated. Figure 4b illustrates the experimental setup, wherein the water-filled cuvette contains a 1 mm diameter column positioned within the object to be grasped. Thereafter, the program enters the object pick-up/release state, whereby the object on the column is manually maneuvered towards the droplet using the micropositioning stage. Figure 12 shows the image sequence corresponding to a ellipsoidal object of about 870 μm pick-up and release experiment, illustrating the stabilized droplet and the object within the grasping column. Subsequently, the object is captured by the microgripper droplet. A similar experiment was conducted using a ellipsoidal object with a tail structure (see Figure 13). While the objects were held by the silicone oil droplet, attempts were made to dislodge them using lateral shearing motions via the column holding the gripper. These attempts were consistently unsuccessful, demonstrating the significant holding strength and stability provided by the established capillary bridge, which is advantageous for secure transport against potential disturbances (Figure 14). 

To evaluate the adaptability of the microgripper, further experiments were conducted with a range of objects possessing different shapes and sizes. These included a 1 mm^3^ cube, a pyramid with a 1 mm^2^ base, a highly irregular asymmetric object, and a series of ellipsoids with principal semi-axes varying from 0.5 mm × 0.4 mm up to 3.0 mm × 2.4 mm.

**Figure 15:**
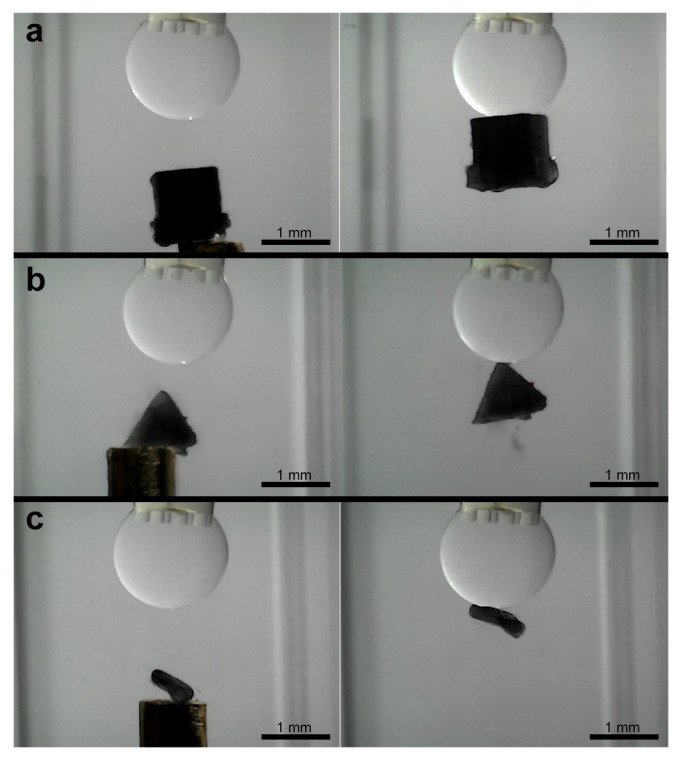
Sequence of capturing of different shapes objects. (**a**–**c**) Cube, pyramid, and irregular shape objects.

**Figure 16:**
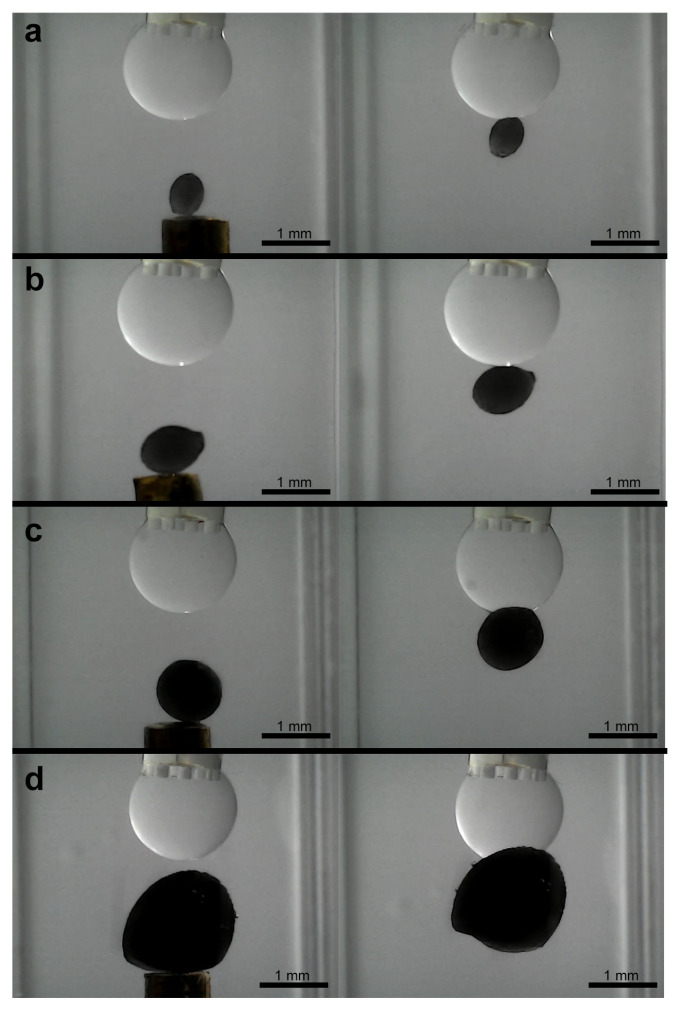
Sequence of capturing of different ellipsoidal objects. (**a**) 0.75 mm × 0.6 mm, (**b**) 1 mm × 0.8 mm, (**c**) 1.25 mm × 1 mm, and (**d**) 2 mm × 1.6 mm ellipsoidal objects.

Successful capture was demonstrated for the different regular shapes, including the cube and pyramid (Figure 15a,b). The irregular object, despite its asymmetry, could also be reliably picked up in any of its different positions (Figure 15c). For the ellipsoidal objects, stable capture were achieved across the tested size range from 0.5 mm × 0.4 mm to 3.0 mm × 2.4 mm (examples shown in Figure 16).

**Figure 17:**
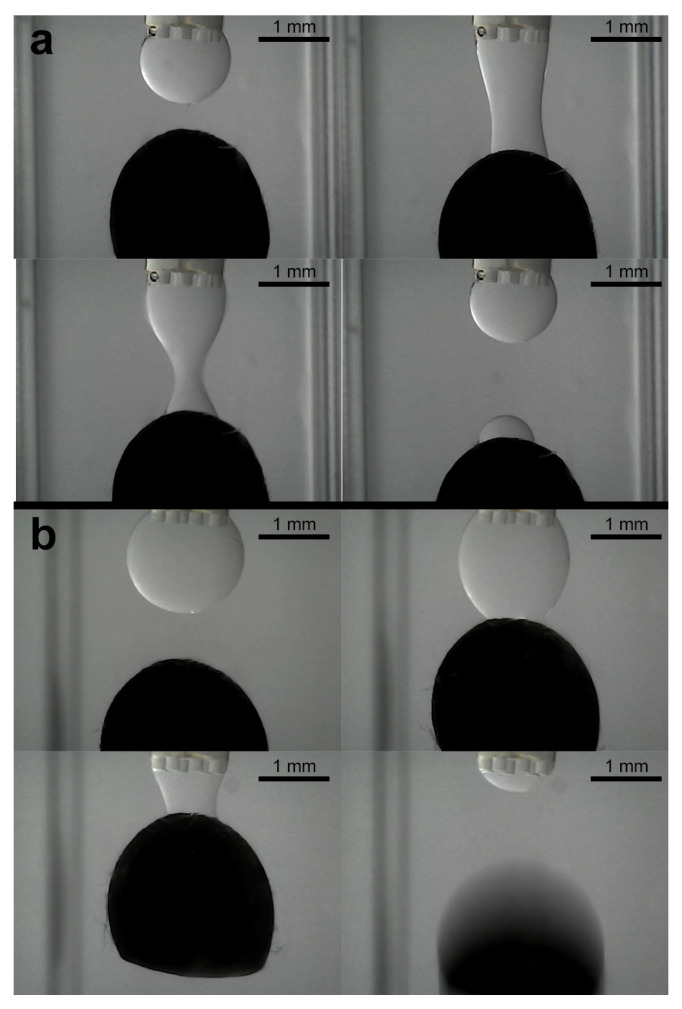
Sequence of tries capturing of largest ellipsoid (3.0 mm × 2.4 mm), illustrating the critical role of adequate droplet volume for successful lift-off and subsequent manipulation. (**a**) Lower droplet volume with unsuccessful capture and (**b**) higher droplet volume with successful capturing and final releasing.

The manipulation of the largest (3.0 mm × 2.4 mm) ellipsoid particularly highlighted the critical role of droplet volume adjustment. When an attempt was made to capture this large ellipsoid using a droplet volume slightly smaller than that typically employed for the baseline or other smaller ellipsoids, the capillary bridge formed but was insufficient to lift the object, breaking before successful capture (Figure 17a). However, by increasing the droplet to a level comparable to or slightly larger than that used for the baseline ellipsoids, the 3.0 mm object could be successfully captured and lifted. This capability to modulate the droplet volume is therefore essential for accommodating a diverse range of object dimensions.

Investigating the release of captured objects revealed several key behaviors. Following simple droplet retraction using the syringe pump, complete release was often not achieved for many of the objects. Objects frequently remained adhered to the gripper tip after the bulk of the silicone oil was withdrawn (Figure 12d and Figure 13d). This persistent adhesion is attributed to the thin, non-volatile silicone oil film remaining on the inner surfaces of the microgripper.

Interestingly, a different release behavior was observed for the largest ellipsoid when captured with a standard or larger volume droplet. For this object, release often occurred relatively easily during the initial phase of droplet volume retraction (Figure 17b). As the droplet volume was reduced, the thinning capillary bridge would break, leading to detachment. This suggests that for larger objects, the balance between the gripping force and the object weight is more delicate, potentially enabling a more passive release mechanism during volume reduction.

**Figure 18:**
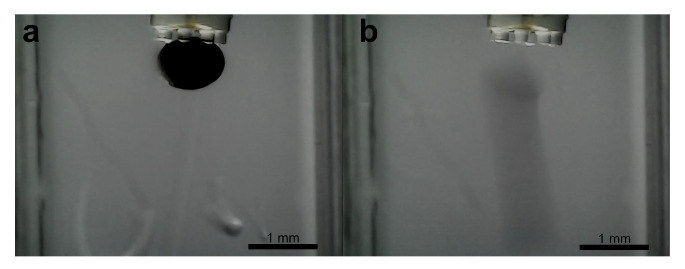
Successful object release using active water flushing. Image sequence showing (**a**) application of external water flow (flushing), and (**b**) detached object released from the gripper. Image contrast is enhanced for improved visualization of the flushing effect.

For objects that remained adhered after complete droplet retraction, alternative active release strategies were necessary. Specifically, inducing a lateral shearing motion and employing active water flushing within the cuvette were explored. Both approaches demonstrated significantly improved release success, reliably detaching the adhered objects from the residual film (Figure 18). The efficacy of these methods likely stems from their ability to directly counteract or circumvent the tensile adhesion forces of the thin oil film: lateral shearing applies a force perpendicular to the main adhesion vector, while active water flushing introduces external hydrodynamic forces. This success with active methods for residual film detachment contrasts sharply with the inability of similar shearing forces to dislodge objects held by the main, intact capillary bridge (Figure 14), further underscoring the difference in force magnitudes.

While these active release methods were implemented manually in the current study, their demonstrated efficacy in dislodging objects from the residual film is particularly relevant for our envisioned application of delivering and releasing micro-scale payloads from a catheter tip within the bloodstream. In such a dynamic in vivo environment, the primary goal is successful deployment within a target region rather than sub-micrometer placement onto a fixed substrate, making these robust detachment techniques highly promising.

In contrast to contact-based compliant grippers like [26], our capillary method avoids direct mechanical stress during capture, and provides a strong, stable hold via the main droplet, resistant to external disturbances like shearing. However, the use of non-volatile oil introduces the specific challenge of overcoming residual film adhesion during release, necessitating the development and optimization of active detachment strategies like shearing or flushing. Our microgripper operates on the same fundamental principle of capillary adhesion governed by the Young–Laplace equation as conventional capillary microgrippers, which typically function by forming a liquid bridge in an air environment [5,6,7,14,27]. These systems effectively leverage the high surface tension of liquids in air to achieve non-contact, low-damage micromanipulation [3,9]. The inherent self-alignment properties derived from minimizing surface energy are also a shared advantage [10,11,12]. However, the critical distinction and primary novelty of our work lie in the adaptation of this principle for stable operation within an aqueous environment. This necessitates the use of a two-phase, immiscible liquid system (silicone oil forming a bridge within water) instead of the conventional water–air approach. This fundamental difference introduces several unique advantages and challenges compared to prior art:

1. Expanded operational domain: The most significant advantage is the ability to operate directly in aqueous media. This opens up applications in biological sample handling, culture media, underwater microassembly, or manipulation in other liquid environments where air-based capillary grippers are inherently unsuitable due to the lack of a stable liquid–gas interface and the high surface tension of the surrounding medium.
2. Stability and elimination of evaporation: Conventional grippers using volatile fluids like water in air face limitations due to evaporation, which alters droplet volume, changes capillary forces unpredictably, and limits the operational time [13]. Our use of non-volatile silicone oil completely circumvents this issue, enabling stable, long-term operation and facilitating the precise, feedback-controlled volume adjustments demonstrated in our experiments.
3. Interfacial tension versus surface tension: The driving force in our system is the interfacial tension between silicone oil and water, which is notably lower than the surface tension of water in air [21]. While this might suggest inherently lower maximum gripping forces, the net force required to hold an object in an aqueous environment is also influenced by buoyancy, which counteracts gravitational forces. Furthermore, our results show that the combination of this interfacial tension and the ability to dynamically control the bridge volume, and thus the contact area/angle, provides sufficient adhesion for securely capturing and manipulating the target microobjects as evidenced by the resistance to shearing forces during capture. Dynamic volume control thus becomes a crucial tool to compensate for or tune forces derived from lower interfacial tension compared to air-based systems.
4. Release mechanism: The non-volatility, while beneficial for stability, introduces a distinct release challenge compared to systems where controlled evaporation can be used as a passive release mechanism [5,6]. The residual oil film necessitates active detachment strategies (like shearing or flushing) after droplet retraction. This contrasts with air-based systems where simple bridge breaking or evaporation might suffice, representing a key design trade-off for achieving aqueous operation. An exception to this was observed during the manipulation of the largest ellipsoid. This suggests that for objects near the upper size limit of the gripper capability, the force balance can become more sensitive to volume changes, potentially offering a passive release pathway that is less prevalent with smaller objects where residual film adhesion dominates more strongly.

## 4. Conclusions

This work has successfully demonstrated the design, fabrication, and experimental validation of a novel capillary microgripper system engineered for stable micromanipulation within an aqueous environment, offering particular promise for challenging tasks such as intravascular delivery via catheters. The core innovation lies in the integration of dynamic droplet volume control using a non-volatile fluid (silicone oil) with vision-based feedback. This enables precise adaptation for capturing a diverse range of object shapes and sizes, overcoming the evaporation limitations inherent in conventional air–liquid capillary systems. The closed-loop control system, utilizing image segmentation and stepper motor actuation, effectively regulated the silicone oil droplet volume, achieving stable positioning crucial for reliable object capture, although with relatively slow response dynamics inherent to the current setup. Simulations performed using COMSOL Multiphysics corroborated the feasibility of forming stable capillary bridges at close proximity, aligning with experimental observations. Experiments confirmed the gripper ability to securely capture microobjects in water, leveraging the interfacial tension between silicone oil and water. The strength of the intended capillary grip was demonstrated by its resistance to lateral shearing forces. However, the use of non-volatile silicone oil introduced a significant challenge during the release phase due to adhesion from residual oil films after droplet retraction. While simple fluid retraction for release was inconsistent for most objects, an exception was observed with the largest ellipsoid, where passive release occurred during initial droplet retraction due to the force balance. For objects remaining adhered, this work further demonstrated that active detachment strategies, specifically lateral shearing or active water flushing, were successful in overcoming this residual adhesion. Therefore, this research validates a robust method for extending controlled capillary manipulation to aqueous applications, offering enhanced stability and adaptability compared to previous approaches.

While the simplicity of the implemented on/off controller with deadband proved sufficient for demonstrating the principle in this study, future work will evaluate alternative control strategies, such as Proportional–Integral–Derivative (PID) control, to potentially achieve faster response times and minimize steady-state oscillations observed in some operating regimes. Also, automating the object approach phase using vision-based control of the micropositioning stage, and developing and automating the identified successful release mechanisms (shearing, flushing) to achieve a fully functional pick-and-release cycle, are key for future works. Force measurements could be carried out to better understand the limits of the microgripper. Further investigations into surface modifications of the microgripper, such as polyethylene glycol (PEG) coating, or alternative non-volatile fluids could also mitigate the residual adhesion challenge. Also, a quantitative assay of chemical or physical contamination of the manipulated objects will be necessary for specific sensitive applications. Overall, this variable-volume, two-phase capillary microgripper presents a significant advancement for applications requiring the precise, stable manipulation of diverse objects in aqueous media, particularly in biomedical engineering and underwater microassembly.

## Figures and Tables

**Table 1:** Performance metrics of the on/off position control system at varying reference positions.

Reference Position (px)	Initial Position (px)	Steady-State Error (px)	Oscillation Amplitude (px)	Oscillation Period (s)	Reference-Normalized ISE
250	108	0.0500	7.1841	32.5364	15.3340
230	181	−1.0000	3.1587	20.2985	1.1491
150	227	0.0500	28.2167	27.6115	13.6832
150	202	−0.3000	19.6545	23.8660	5.1483
115	148	−0.4500	7.5858	20.3094	1.8236

## Data Availability

The original contributions presented in this study are included in the article. Further inquiries can be directed to the corresponding authors.
